# The Second Inter-Allied Conference

**Published:** 1918-06-08

**Authors:** 


### THE AFTER-CARE OF DISABLED MEN.

### The Second Inter-Allied Conference.

By A SOCIAL WORKER.

The first Inter-Allied Conference on this subject was held in Paris last year, the second has just been held in the Central Hall at Westminster. It was opened and closed by the Duke of Connaught, and the exhibition connected with it was visited by the King and Queen and other members of the Royal Family. It is, however, to be regretted that the general public were not more fully aware of the extreme interest and value of this exhibition. We are glad that the plan mentioned at the closing ceremony by Mr. Hodge has been carried out, and another hall secured, so that the collected treasures will be shown in London from June 3 to 15 at the Memorial Hall, Farringdon Street; 6d. admission—free to Service and silver-badged men. It will then be sent to some provincial cities. Such a step will certainly do something to solve the problem which the discussions of the conference showed to be equally pressing in all countries, that of securing for disabled men the re-education which will make them independent of charity and securing also their acceptance of the opportunities offered.

### Some of the Exhibits.

There are innumerable exhibits which show the successful conquering of extreme difficulty. For instance, a lectern—and also a tray with carved border completed during the week—was made by Private G. Pell, who is blinded, and had had no experience of carpentry till he went to St. Dunstan's. A meat-safe with zinc panels was made by Lance-Corporal Hopper, who is not only blind but has lost the first finger of the right hand, and thumb and first finger of left hand. Weaving has been taken up, especially by the Canadians, who are also sending their disabled men largely to established schools for improvement in general education. Some beautiful silk weaving on small portable looms is shown by them, and in more than one case men with arms more or less disabled, and whose previous occupations had been such as mining and engine-driving, had made up their weft into articles of women's clothing finished with chiffon trimmings and hand-made tassels. Similarly, a pair of ladies' boots shown by the Port Villez Belgian Training Centre were the combined work of a gardener, a mason, and a miner, but had every appearance of highly-trained skill. Indeed, much latent talent has been drawn out. The Italian bookbinding in tooled and gilt vellum and morocco, their leather cushions, and the leather backs and seats added to some of their finely designed and carved chairs would be remarkable anywhere.

### Furniture, Stained Glass, and Doll-Modelling.

The New Zealanders in their convalescent camp at Hornchurch, Hants, should have a good market for their stained glass, their hammered pewter set with coloured stones, and the dyed raffia which is so admirably used as Bargello embroidery for furnishing purposes. Furniture, indeed, comes in great variety and on practical lines from many places, not least our own "Lord Roberts'" workshops. The exhibits of this centre are of great variety, perhaps not least important is the now admirable and not highly priced doll-modelling which gives real hope of a capture of this important industry.

Prisoners in Switzerland show a fine carpet, and piano parts. Metal-work is noteworthy in the engineering section, and boot repairs from St. Dunstan's and the Cordwainers' College. The leather bags made at Mare Street, Hackney, and elsewhere, are lined with silk and finished in a way which would do credit to far longer training. The French raised pictures for training blind masseurs, poultry-keepers, etc., are very interesting, as also the collection of copy-books and note-books showing that in all countries efforts are being successfully made to enable a man to profit by his past experience with the addition of new knowledge in order to take a higher post in his former occupation. Thus a shop salesman, when taught book-keeping and a knowledge of textiles and so forth, may be placed as secretary to a managing director.

### Economic, Physical, and Financial Difficulties.

Re-education, therefore, is seen to be of several kinds. First, if necessary, the raising of the man's general knowledge; in Italy the excellent rule prevails that each discharged man must attend school for a fortnight at least. Secondly, the learning of another form of his own craft, or a craft now suited to his condition. The supreme importance of this was emphasised in many papers and speeches during the conference, and the need of an adequate supply of skilled teachers was dwelt upon by Sir C. Nicholson in summing up the discussions of the Training Section. The economic difficulty of the competition of a man with a pension which may be exploited by employer or employee in case of industrial strife, the physical difficulty of his competition with the sound, and the financial difficulties of his output were much discussed. "These," said a Portuguese delegate, speaking of the most grievous cases, "will throughout life continue to make the supreme sacrifice." "In France," said a member of the French Chamber, "there will be few entirely sound men in any employment." The further difficulty of this question of training lies in the need of sympathetic workers who will persuade men not to fall back into pensioned idleness, but to persevere, despite any hindrances of pain or depression, till knowledge is won. Hence, thirdly, the need of re-education in the use of an injured body and the appliances designed to keep it. A photograph in the French section shows a man blinded and with both hands gone working a knitting-machine by the aid of two affixed hooks. Much experience already goes to show that the using of artificial limbs, however exquisitely made—and Mr. Myers, of Roehampton, told the conference that every day sees fresh improvements—is in itself a craft and not an entirely easy one. In a speech which many delegates declared to be one of the best of those heard throughout the week, Mrs. Wood, of the London War Pensions Committee, pleaded earnestly for long-continued care of men after their apparently satisfactory establishment in industry.

### Small Holdings for the Tuberculous.

There remain the men disabled by illness, and naturally the case of the tuberculous attracted much attention. The question of whether *post hoc* would be *propter hoc* in all cases, the later development of tubercle and in what proportion was discussed more than once. The objection to agricultural work for such cases, in England at least, authoritatively made, as it was, gives food for thought. "The work is apt to be too heavy, housing is bad, food often unsatisfactory." An extremely valuable suggestion, however, is that small holdings should be combined with the much-needed re-afforestation of these islands. Forestry offers healthy and not over-severe work under the right conditions for such cases, and occupation in winter. One result, no doubt, of the increased unity of purpose produced by such conferences will be seen in the application of climate to early stages of tuberculous trouble.

